# Novel antibiotic combinations proposed for treatment of *Burkholderia cepacia* complex infections

**DOI:** 10.1186/s13756-017-0279-8

**Published:** 2017-11-25

**Authors:** Omar M. El-Halfawy, Marwa M. Naguib, Miguel A. Valvano

**Affiliations:** 1 Department of Microbiology and Immunology, University of Western Ontario, London, ON Canada; 2 Department of Microbiology and Immunology, Faculty of Pharmacy, Alexandria University, Alexandria, Egypt; 3 Wellcome-Wolfson Institute for Experimental Medicine, Queen’s University Belfast, Health Sciences Building, 97 Lisburn Road, Belfast, BT9 7BL UK; 4 Department of Microbiology and Immunology, Faculty of Pharmacy, Damanhour University, Damanhour, Egypt

**Keywords:** *Burkholderia cepacia* complex, Moxifloxacin, Ceftazidime, Colistin, Cystic fibrosis, Antibiotic combinations

## Abstract

Effective strategies to manage *Burkholderia cepacia* complex (Bcc) infections in cystic fibrosis (CF) patients are lacking. We tested combinations of clinically available antibiotics and show that moxifloxacin-ceftazidime could inhibit 16 Bcc clinical isolates at physiologically achievable concentrations. Adding low dose of colistin improved the efficacy of the combo, especially at conditions mimicking CF respiratory secretions.

## Introduction

Respiratory failure secondary to chronic pulmonary bacterial infection remains the primary cause of mortality and morbidity in cystic fibrosis (CF) patients [1]. We investigated the efficacy of non-standard antibiotic combinations to combat multidrug resistant *Burkholderia cepacia* complex (Bcc) bacteria. Bcc comprises a group of closely related species of which *B. cenocepacia*, *B. multivorans*, and *B. contaminans* are frequently isolated from CF patients [2, 3]. Bcc infections cause faster decline in lung function [4] and severely hinder post lung transplant survival of CF patients [5–7].

Effective management strategies are lacking for Bcc eradication in CF [8]. The EUCAST and the BSAC no longer provide recommendations for antimicrobial susceptibility testing against Bcc, while CLSI provides guidelines for seven agents to test for therapeutic use against Bcc [9]. These include three β-lactams (ceftazidime, meropenem, and ticarcillin-clavulanate), the fluoroquinolone levofloxacin, and trimethoprim-sulfamethoxazole combo (co-trimoxazole) in addition to the bacteriostatic drugs minocycline and chloramphenicol. New therapeutic solutions are being explored [10–12], but until they can be translated into clinical use, Bcc-infected patients are in dire need of effective therapeutics. We aimed to bridge this gap by finding novel combinations of clinically available antibiotics that could eradicate Bcc bacteria at physiologically relevant concentrations and could be readily used in patients. We focused on bactericidal antibiotics for which CLSI guidelines exist as candidates for combination therapy, avoiding previously tested combinations that showed no synergy against Bcc [13–15].

## Methods

*B. cenocepacia* K56–2 was isolated from a CF patient in Canada and obtained from the *B. cepacia* Research and Referral Repository for Canadian CF Clinics (BCRRC); it is commonly used as a prototypic strain of the *B. cenocepacia* ET-12 epidemic clonal lineage [16]. A panel of 6 *B. cenocepacia*, 5 *B. multivorans* and 4 *B. contaminans* strains were isolated from CF patients. Bacteria were cultured in Luria-Bertani (LB) or Mueller-Hinton broth (MHB) media at 37 °C. LB is commonly used to grow Bcc isolates in our laboratory, while MHB is the recommended medium for standard antimicrobial susceptibility testing. MIC was initially determined by Etest strips (BioMérieux Inc., St. Laurent, Qc, Canada) as previously described [17].

Checkerboard assays were conducted with combinations of antibiotics (obtained from Sigma, St Louis, MO, USA) as previously described [10]. Initial assays against *B. cenocepacia* K56–2 were conducted in LB medium to select the most potent combination. Subsequent checkerboard assays of moxifloxacin-ceftazidime in the presence or absence of 4 μg/ml colistin sulphate against a panel of *B. cenocepacia*, *B. multivorans* and *B. contaminans* clinical isolates were conducted in MHB as recommended by CLSI for MIC testing by broth microdilution [18]. When accurate MIC values could not be determined, as for colistin methanesulfonate because Bcc bacteria grow at concentrations greater than its solubility in growth medium, the highest concentration tested was considered to be half the MIC value. Fractional inhibitory concentration indices (FICI) were calculated as FICI = A/MIC_A_ + B/MIC_B_, where A and B are the concentrations of two antibiotics required in combination to inhibit bacterial growth and MIC_A_ and MIC_B_ are the MIC values for drugs A and B alone [19]. FICI data were interpreted as ‘synergy’ (FICI ≤ 0.5), ‘antagonism’ (FICI > 4.0), and ‘no interaction or indifference’ (FICI 1–4.0).

Artificial sputum medium (ASM) mimicking CF sputum was prepared as described [20] with the exception that components of the medium were autoclaved, filter-sterilized, or obtained already sterilized (instead of adding antibiotics). 20 mg/ml mucin (instead of 10 mg/ml) was added according to Quinn et al. [21]. Overnight cultures of *B. cenocepacia* K56–2 in LB medium were diluted in sterile ASM with or without antibiotic(s) to reach an inoculum equivalent to OD_600_ of 0.004 (~10^6^ CFU/ml) and incubated at 37 °C without shaking. Bacterial growth was assessed by CFU count on LB agar plates at different time points.

## Results and discussion

**Fig. 1:**
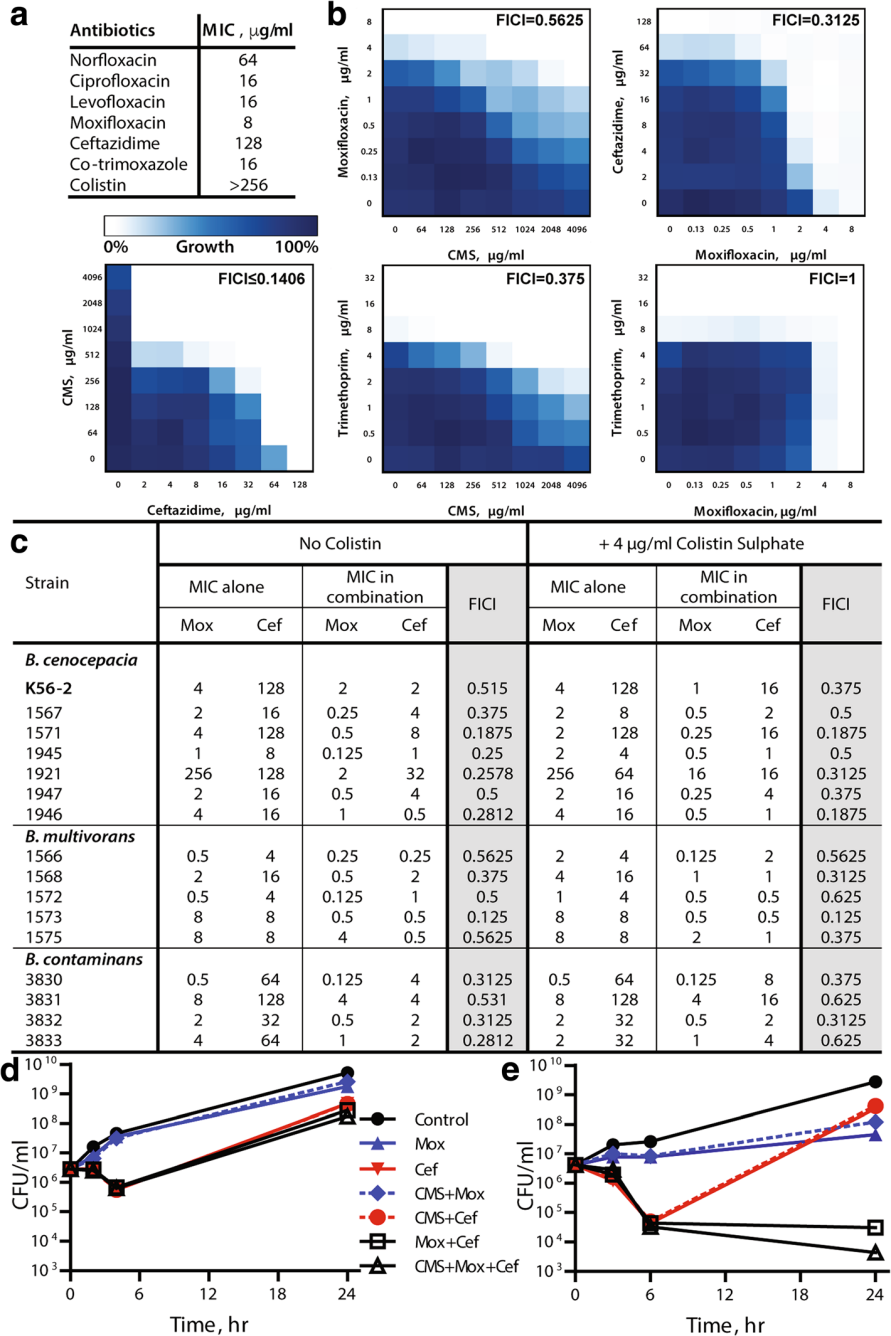
Antibiotic combination testing against Bcc bacteria. **a** MIC by Etest against K56–2 at 24 h. **b** Checkerboard assays in LB medium against K56–2. **c** Checkerboards assays in MHB per CLSI microbroth dilution method against a panel of Bcc. **d** and **e** CFU counts in ASM; mean ± SEM, *n* = 4 from 2 independent experiments. Antibiotic concentrations are: (**d**) 8 μg/ml ceftazidime (Cef), 2 μg/ml moxifloxacin (Mox) and 10 μg/ml CMS; and (**e**) 16 μg/ml Cef, 10 μg/ml Mox and 20 μg/ml CMS

The MIC of individual antibiotics was first determined by Etest against *B. cenocepacia* K56–2 prior to combination testing. We tested ceftazidime, a representative β-lactam antibiotic that showed success in inhaled formulations for treating *P. aeruginosa* lower respiratory tract infections in CF patients [22], and has activity against *B. cenocepacia* [14, 17, 23]. Ceftazidime showed an MIC of 128 μg/ml against K56–2 (Fig. 1a). We tested levofloxacin and other fluoroquinolones from different generations; K56–2 displayed lower resistance levels to them, with norfloxacin being the least potent (MIC = 64 μg/ml) relative to the tested newer generation agents especially moxifloxacin (MIC = 8 μg/ml) (Fig. 1a). Co-trimoxazole showed an MIC of 16 μg/ml against the prototypic *B. cenocepacia* isolate; whereas, K56–2 was highly resistant to colistin (MIC >256 μg/ml) (Fig. 1a). Despite its lack of activity against Bcc, colistin was included in the study owing to its reported ability to permeabilize the cell envelope of Gram-negative bacteria to other antibiotics [24, 25]. Notably, the MIC of the tested antibiotics against K56–2 are above the CLSI clinical breakpoints for susceptibility.

Next, we conducted checkerboard assays for select combinations against *B. cenocepacia* K56–2. Colistin methanesulfonate (CMS) showed borderline synergism with moxifloxacin (Fig. 1b). CMS is a less toxic prodrug of colistin that is active in vitro and in vivo [26, 27]; 4 μg/ml of CMS is equivalent to 1.5 μg/ml of colistin base activity [28]. The trimethoprim-CMS combination was synergistic whereas trimethoprim-moxifloxacin showed indifference (Fig. 1b). Furthermore, ceftazidime combinations with either moxifloxacin or CMS were synergistic (Fig. 1b). Since the combination of moxifloxacin and CMS was also synergistic, these 3 antibiotics (ceftazidime, moxifloxacin and CMS) were chosen for further follow-up testing.

We further tested whether the synergistic effects of these three antibiotics against K56–2 are reproducible against other clinical isolates of Bcc bacteria and in MHB following the CLSI guidelines. Checkerboard assays of ceftazidime-moxifloxacin combinations showed similar synergistic patterns against a panel of 7 *B. cenocepacia*, 5 *B. multivorans* and 4 *B. contaminans* in MHB (Fig. 1c). Such synergism remained, or even further increased in some cases, in the presence of 4 μg/ml colistin sulphate (Fig. 1c). More importantly, these antibiotic combinations inhibited the Bcc clinical isolates at or below the clinical breakpoints set by CLSI when in combination but not individually in most cases (Fig. 1c). The CLSI breakpoints are 8 μg/ml for ceftazidime; and 2 μg/ml for levofloxacin, which is closely related to moxifloxacin, for Bcc [9]. Therefore, this shows promise that triple combination of these antibiotics would eradicate Bcc at clinically achievable concentrations.

To test the efficacy of these combinations in CF sputum-like conditions, we used an artificial CF sputum medium (ASM) and determined the CFUs of K56–2 at different time points following treatment with the antibiotic combinations. Low antibiotic concentrations, equivalent to the CLSI breakpoints where available (8 μg/ml ceftazidime, 2 μg/ml moxifloxacin and 10 μg/ml CMS), resulted in killing of only ~1-log at the 4 h time-point compared to the initial inoculum (up to 2-log less than the untreated control at the same time-point) in ASM (Fig. 1d). Reduced antibiotic efficiencies in ASM compared to LB and MHB is not unexpected given the reported lack of correlation between in vitro susceptibility testing results performed in standard laboratory media as MHB and the clinical outcome in CF patients [29]. Therefore, higher antibiotic concentrations were tested in ASM (Fig. 1e). Moxifloxacin (at 10 μg/ml) had a bacteriostatic effect with no appreciable change in survival over 24 h compared to the initial inoculum (2-log reduction in CFU compared to control at 24 h). Ceftazidime at 16 μg/ml resulted in significant initial killing of 2-log CFU from the starting inoculum (up to 3-log CFU reduction relative to the control at 6 h). However, overgrowth of resistant cells occurred at 24 h leading to only 1-log reduction compared to control values at the same time-point and an increase of almost 2-log CFU relative to the initial inoculum. Combining moxifloxacin with ceftazidime prevented overgrowth of resistant cells and sustained the killing effect of ceftazidime until 24 h (5-log reduction compared to the untreated control at 24 h). CMS at 20 μg/ml further reduced the CFU by 1-log (3-log CFU killing relative to the initial inoculum or 6-log total CFU reduction compared to untreated control at 24 h) when combined with moxifloxacin and ceftazidime (Fig. 1e). These concentrations, although slightly above the CLSI breakpoints, are physiologically achievable in respiratory fluids and tissues (see FDA documents available for moxifloxacin [30] and for ceftazidime [31]).

In summary, we report novel double and triple antibiotic combinations that inhibit Bcc bacteria at physiologically achievable concentrations and hence could be ready for immediate use in patients. In addition, nanotechnology-based novel respiratory delivery systems may deliver even higher doses of these antibiotics at the local site of infection. For example, a pilot trial of long-term administration of tobramycin inhalation powder delivered using a Podhaler has shown some promise for CF patients chronically infected with Bcc [32], despite the low efficiency of tobramycin against Bcc in vitro [33]. We then propose these combinations as ideal targets for experimental screening of novel antibiotic adjuvants for enhanced efficacy against Bcc bacteria.

## Abbreviations

EUCAST: European Committee on Antimicrobial Susceptibility Testing
BSAC: British Society of Antimicrobial Chemotherapy
CLSI: Clinical and Laboratory Standards Institute
